# Acute septic arthritis of the acromioclavicular joint caused by Staphylococcus aureus with marked soft tissue collection towards posterior medial aspect of the AC joint: A rare clinical presentation^☆^

**DOI:** 10.1016/j.idcr.2022.e01513

**Published:** 2022-05-24

**Authors:** Jija Thomas, Makki Daud, Simon Macmull

**Affiliations:** a Dudley NHS Trust Foundation, UK; b NHS West Hertfordshire, UK

**Keywords:** Acromioclavicular joint, Septic arthritis, Infection, Magnetic resonance imaging, Staphylococcus aureus

## Abstract

Primary septic arthritis of the Acromioclavicular joint is an unusual disorder and is seldom seen even in an immunocompromised person. We report a case of primary septic arthritis of the acromioclavicular (A-C) joint caused by Staphylococcus aureus. The patient was admitted with pain in the left shoulder, restricted movements and fever. Laboratory parameters showed elevated C-reactive protein, raised erythrocyte sedimentation rate and leukocytosis. Ultrasound revealed a 32 mm collection at the acromioclavicular joint. Patient underwent incision and drainage of abscess. Culture and sensitivity revealed moderate growth of Staphylococcus-aureus. Patient was started on appropriate intravenous antibiotics. Magnetic resonance imaging (MRI) done after 2 weeks revealed marked erosion in the lateral end of clavicle with soft tissue collection along the posteromedial aspect A-C joint. The patient had to undergo repeat drainage of the abscess along with the decompression of lateral end of clavicle. The patient was successfully treated with 8 weeks of appropriate antibiotics with complete resolution of infection.

## 1 Introduction

Septic arthritis of the acromioclavicular (AC) joint is a rare entity with symptoms that include fever, erythema, swelling, tenderness over the AC joint and limitation of shoulder motion with pain. The diagnosis of this condition can be difficult and may be confused with degenerative AC arthritis or glenohumeral pathology. This disease is most seen in patients with a preexisting immunocompromised state; however, it has also been reported in patients without any predisposing comorbidities[1], [2], [3], [4], [5], [6], [7], [8], [9], [10], [11], [12], [13], [14], [15]. With an increasing number of patients with compromised immune systems from etiologies such as diabetes, drug abuse, cancer, and chemotherapy, this disease process may become more prevalent in the future. Staphylococcus aureus is the commonest organism isolated in blood or joint aspirate of patients with septic arthritis [2], [9], [13], [16], [17], [18]. Here, we report the case of a patient with septic arthritis of the Acromioclavicular joint with its extension towards posterior medial aspect of the AC joint. There are very few cases in literature which have reported this presentation of acromioclavicular joint septic arthritis.

## 2 Case report

**Fig. 1:**
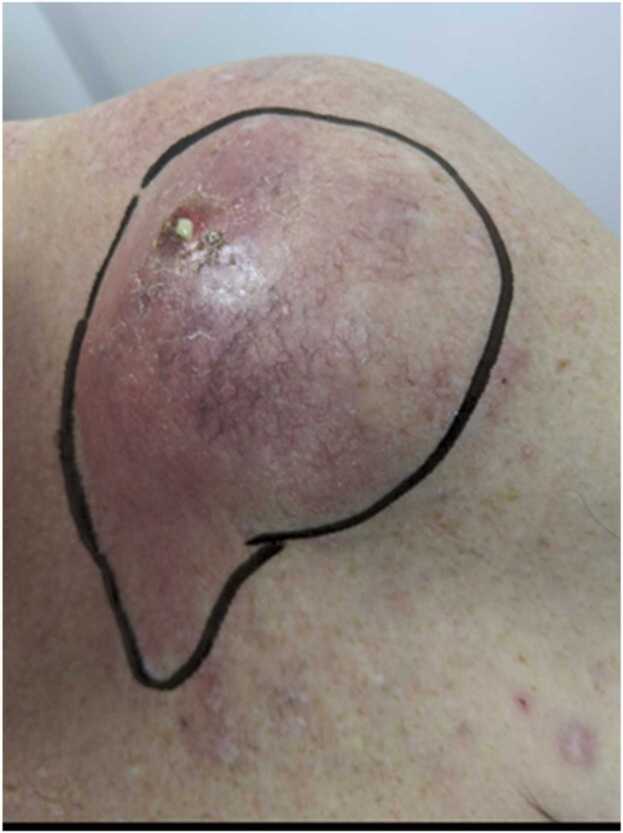
Photograph of the patient when he initially presented.

A 64-year-old right-handed male presented with 2 weeks history of pain, fever, and restricted movements in his left shoulder. There was no history of injury. His past medical history included chronic obstructive pulmonary disease, rheumatoid arthritis and left sided frozen shoulder. He was on Leflunomide, Fostair. Patient is a smoker, allergic to penicillin, grade2 performance WHO (ambulatory and capable of all self-care, but unable to carry out any work activities). On examination, he was febrile. Local examination revealed a 5 cm fluctuant swelling over the A-C joint with surrounding erythema, with 2 discharging punctum and local rise of temperature(Fig. 1)The A-C joint was tender and shoulder movements were restricted. The cross-body adduction test produced pain at the Acromioclavicular joint, internal rotation was intact with 45-degree abduction. There was no vascular or neurological deficit. The rest of the body examination was normal.

**Fig. 2:**
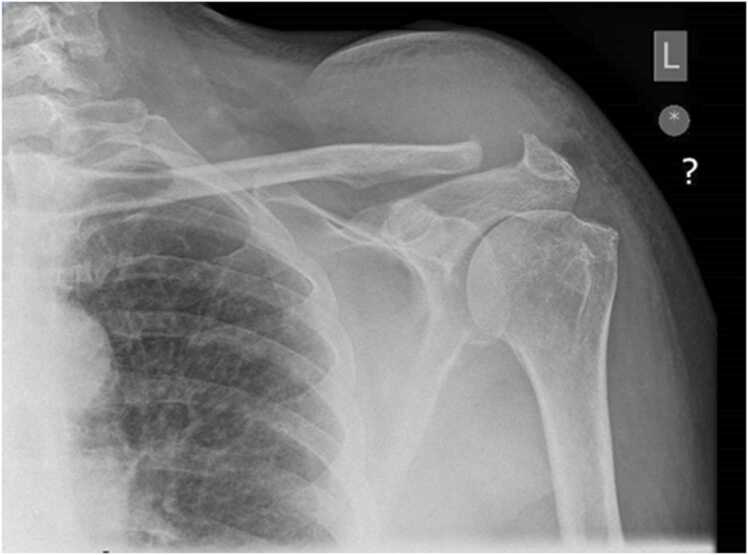
A plain radiograph of the shoulder showed joint space widening with acromioclavicular joint osteoarthritis.

**Fig. 3:**
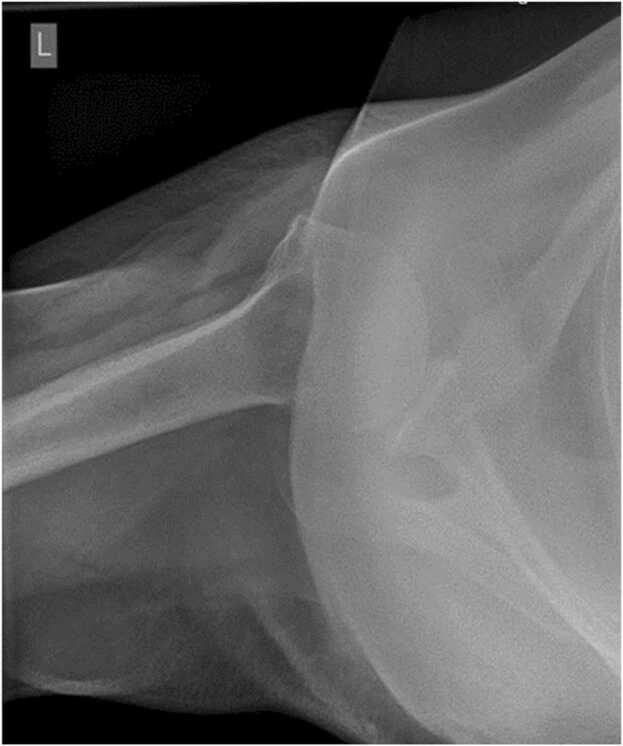
Plain radiograph of the shoulder (Axial view) showing joint space widening and acromioclavicular joint osteoarthritis.

**Fig. 4:**
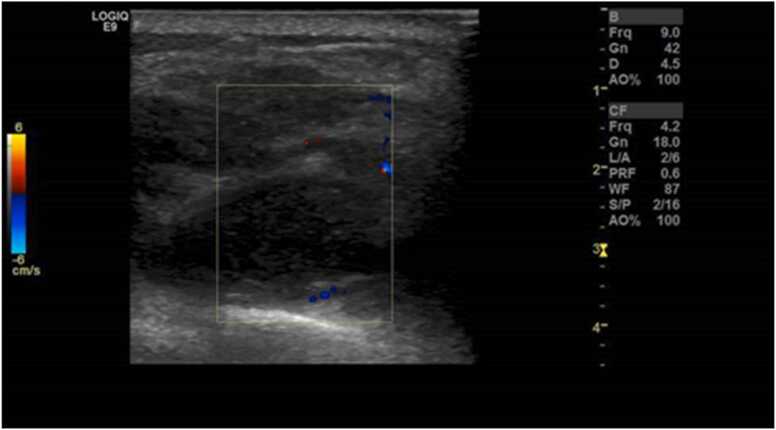
Ultrasonography showed 32 mm fluid collection over the left Acromioclavicular joint, with increased vascularity.

The inflammatory parameters were raised with a C-reactive protein (CRP) of 5.3 mg/L and erythrocyte sedimentation rate (ESR) of 77 mm/h. The white cell count was 13.6 × 10^9^/L with a raised neutrophil count of 11.29 × 10^9^/L. A plain radiograph of the shoulder AP and axial view (Fig. 2 & Fig. 3)showed joint space widening with acromioclavicular joint osteoarthritis. Ultrasonography showed 32 mm fluid collection over the left Acromioclavicular joint, with increased vascularity(Fig. 4).

Abscess drainage was performed by an incision connecting both punctums 0.200 ml of frank pus was drained and sent for culture and sensitivity. Unhealthy skin edges were debrided, cavity was curetted and packed with Kaltostat Alginate. Pus drained from the A-C joint grew moderate growth of Staphylococcus aureus. As patient was allergic to penicillin, he was commenced on parenteral therapy of Teicoplanin 800 mg once daily as per sensitivity. Echocardiography was done to rule out infective endocarditis.

**Fig. 5:**
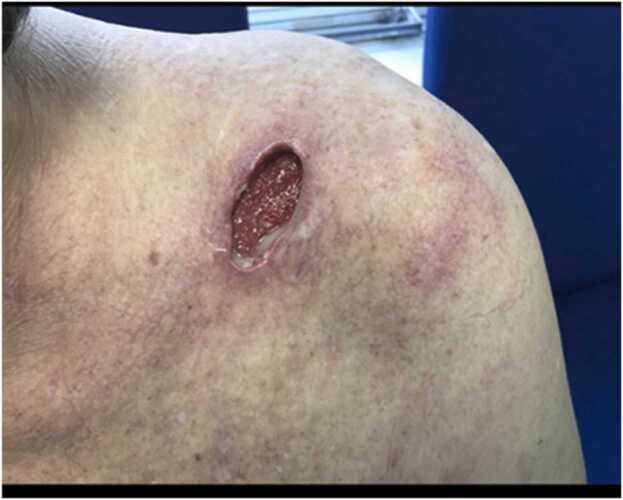
Photograph of the patient after two weeks.

**Fig. 6:**
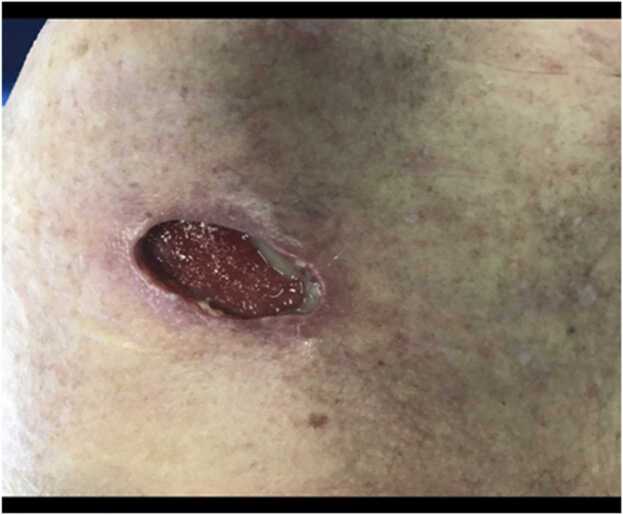
Photograph of the patient after two weeks.

**Fig. 7:**
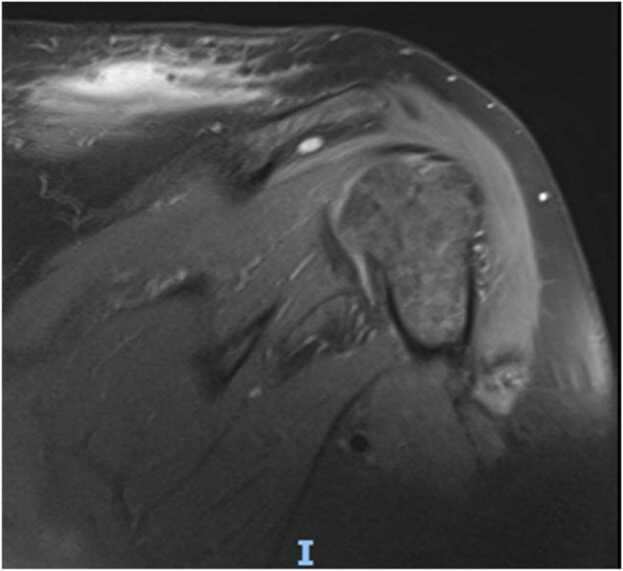
Precontrast and post contrast MRI images of patient performed at 2 weeks.

**Fig. 8:**
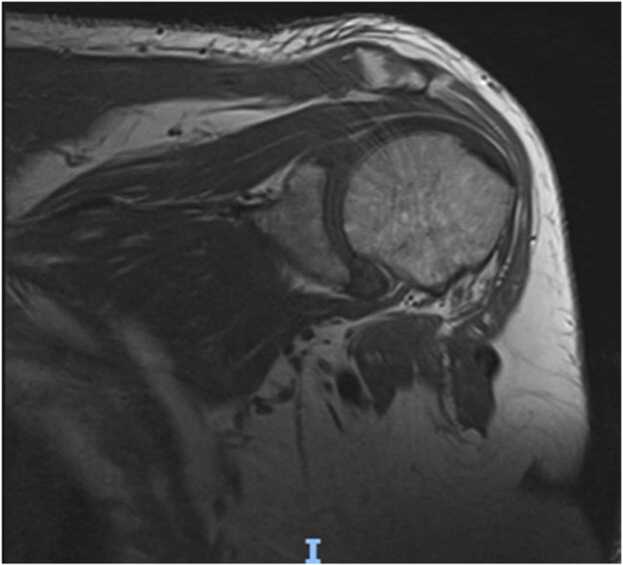
Precontrast and post contrast MRI images of patient performed at 2 weeks.

**Fig. 9:**
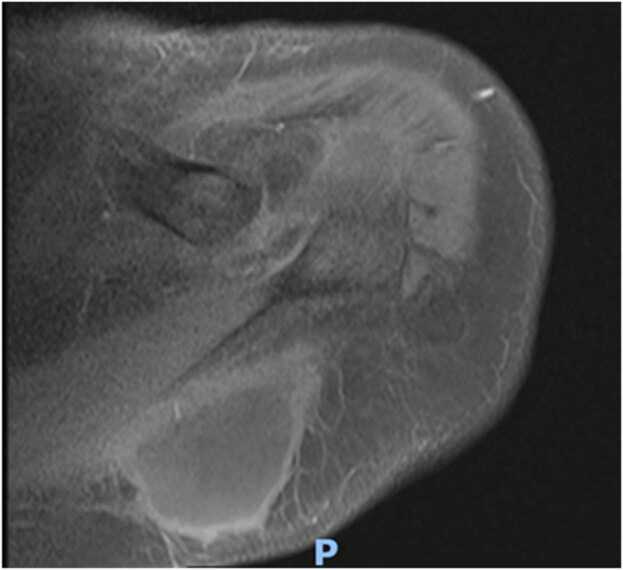
Precontrast and post contrast MRI images of patient performed at 2 weeks.

**Fig. 10:**
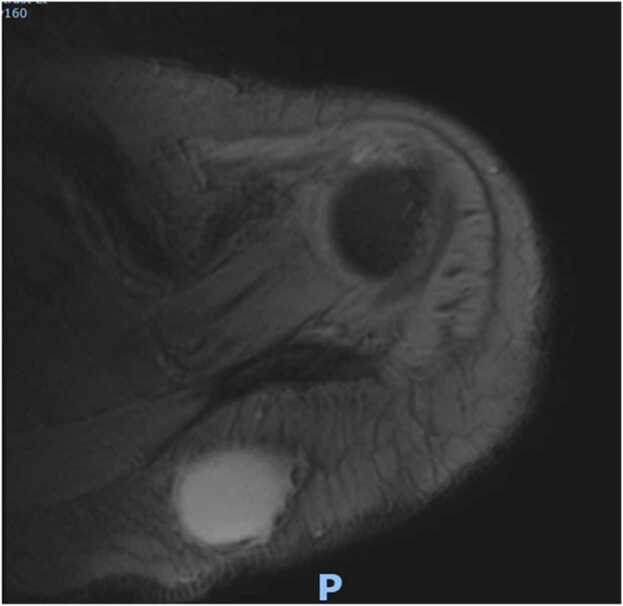
Precontrast and post contrast MRI images of patient performed at 2 weeks.

At two week follow up even though the incision sites were healing well(Fig. 5 & Fig. 6) patient was having persistent raised inflammatory markers. A follow up MRI scan after two weeks with contrast revealed 52×45×32 mm focal collection within the superficial soft tissues towards the posteromedial aspect of the left Acromioclavicular joint, communicating with the left AC joint(Fig. 7 &Fig. 8). The left AC joint was widened and demonstrated moderate effusion, subchondral edema and synovial enhancement on post contrast imaging. Overall features suggested of Left acromioclavicular joint septic arthritis(Fig. 9 & Fig. 10). Mild chondral thinning was noted with in the glenohumeral joint but no significant articular cartilage loss.

Posteromedial abscess drainage was done by extending the previous incision along with decompression of lateral end of clavicle 0.150 ml of frank pus drained and sent for culture and sensitivity. His culture and sensitivity showed scanty growth of Staphylococcus aureus, sensitive to doxycycline. Patient was discharged on oral therapy of Doxycycline 100 mg once daily for a period of three weeks. The inflammatory parameters showed progressive improvement over the treatment period.

He reported no fever during the therapy time and having no exacerbation of pain. He could move his left shoulder with limitation of abduction and external rotation which he previously had due to frozen shoulder. At the 2 month follow-up consultation, the patient was asymptomatic, with some limitation of shoulder motion, the scars were closed without inflammation and with no signs of osteomyelitis on the radiographs.

## 3 Discussion

Septic arthritis most commonly affects larger joints of the body and spread by hematogenous route. Bacterial infections of the joints are usually mono-articular in 85– 90% of cases. The most involved joint is the knee (50%), followed by the hip (20%), shoulder (8%), ankle (7%), and wrists (7%)[6], [9]. Septic arthritis of the AC joint is a very rare condition with an incidence of 2–10 per 100,000 in the general population [6]and more prevalent in men with a mean age of 50–60 years[3]. It can co-exist with shoulder septic arthritis in many cases, so incidence of AC joint septic arthritis alone is unknown [19]. In addition to direct trauma or surgical intervention to the AC joint, the common reported causes of this conditions were immunocompromised conditions such as multiple myeloma [4], acquired immunodeficiency syndrome [13], and long-term steroid use[20]. Chronic systemic diseases are also risk factors for Acromioclavicular joint septic arthritis[6]. The earliest case of septic arthritis of the AC joint was reported by Blankstein et al. [2]in 1985, the patient was treated with antibiotics and surgical drainage.

A good clinical examination is mandatory in the diagnosis of Acromioclavicular joint septic arthritis and can help to differentiate it from glenohumeral arthritis [9]. The shoulder movements can be restricted in active and passive motions. Patients will often have significant tenderness on palpation over the AC joint but may have diffuse tenderness because of surrounding myositis[11]. Cross-body adduction test are often strongly positive in these patients [1], [4], [8], [14]. Due to the small size of the AC joint, there is a high risk of local spread of infection in the surrounding planes, so thorough examination of surrounding planes must be done for any collection[3]. In this case patient was having a collection in the posteromedial aspect of the AC joint which was missed during the initial examination.

Laboratory studies are important in the diagnosis of an infection; however, inflammatory parameters should not be the primary diagnostic tool used, but rather should supplement other diagnostic criteria. Imaging plays a vital role in the diagnosis of a septic arthritis. In an acute presentation, initial findings on plain radiographs may include soft tissue swelling and joint space widening due to localized oedema and effusion[21]. Erosion and destruction occur late in the disease process [6]. Even though Technetium99 and gallium-citrate67 scans are frequently positive with asymmetric uptake in patients with septic arthritis, they lack significant specificity [21]. They cannot distinguish an inflamed joint from an infected joint. Ultrasonography can diagnose Acromioclavicular joint inflammation by detecting distension of the joint and can help as a guide in aspirating the AC joint which can be challenging to aspirate, but it is operator dependent, and is less helpful than MRI [12], [22], [23]. MRI can detect a septic joint as early as 24 h after infection even in small joints with minimal synovial fluid as in the Acromioclavicular joint [6]. As the Acromioclavicular joint is in proximity with the Glenohumeral joint MRI can be particularly useful in excluding any involvement of Glenohumeral joint, integrity of the rotator cuff and surrounding myositis. As in our case (gadolinium enhanced) MRI with fat suppression was particularly useful in diagnosing septic arthritis as well as extension of abscess along the posteromedial plane which was missed initially in the ultrasound and has been reported to be an extremely sensitive test for septic arthritis, with a sensitivity of 100% [24], [25]^.^ MRI also can provide information on the presence or absence of concurrent osteomyelitis which can affect treatment and antibiotic management. It should be obtained in any patient for whom there is uncertainty about the diagnosis, as it can provide the treating with a correct differential diagnosis.

After the diagnosis a suitable treatment regime should be planned. Effective treatment involves surgical irrigation and debridement, or aspiration of the Acromioclavicular joint followed by a course of IV or oral antibiotics [1], [2], [3], [4], [5], [6], [7], [8], [9], [10], [11], [13], [14], [15]. Staphylococcus aureus is the commonest organism isolated in blood or joint aspirate of patients with Acromioclavicular joint infection[9], [13]. Other offending organisms include Streptococcus viridans[2], Streptococcus bovis and Group D Streptococcus[6], Mycobacterium avium-intracellular [26] and Streptococcus pneumoniae[12]. In this case there was no trauma, injury and the main organism involved was Staphylococcus aureus.

Complete debridement of the joint must be done either arthroscopically or open. There is not adequate literature to determine if arthroscopic or open debridement is a better option. Lateral end of clavicle excision or decompression at the time of surgery should be performed if osteomyelitis of the distal clavicle is suspected or if there is co-existent osteoarthritis. Aspiration of the Acromioclavicular joint may be done if patient is not fit for surgical management. If aspiration is performed, the patient should be monitored, and if no improvement is seen clinically or with inflammatory markers, then surgical irrigation and debridement should be considered. During the procedure exploration of the communicating planes must be done for any other abscess collection. Intravenous antibiotics should be used, as this provides better tissue penetration and after adequate control of the infection is obtained, transition to oral antibiotics is appropriate [27]. The average duration of successful antibiotic management in the current literature is 5.9 weeks. This is comparable to our patient who was treated with 6 weeks of antibiotics with resolution of the infection. There are currently no specific guidelines for the duration of treatment for septic arthritis [27]. It is always recommended to consult microbiologist for the choice of antibiotic and duration of treatment in order to maximize quality of patient care.

## 4 Conclusion

In summary, septic AC joint arthritis is a rare condition which is difficult to be diagnosed and can be easily missed. Key to early diagnosis is thorough clinic examination of acromioclavicular joint and planes communicating with it. It should be coupled with laboratory values and advanced imaging studies to make the diagnosis. Treatment can consist of irrigation and debridement or aspiration of the AC joint followed by antibiotic management for at least four to six weeks with guidance from microbiologist if available.

## Consent

Appropriate consent was taken from the patient

## Declaration of Conflicting Interests

The author(s) declared no potential conflicts of interest with respect to the case reporting, authorship, and/or publication of this article.

## Funding

The author(s) disclosed that no financial support was taken from any person for this case report.

## Ethical approval

All of the authors certify that all investigations were conducted in conformity with ethical principles of research.
